# Is Performance-Based Financing A Pathway to Strategic Purchasing in Sub-Saharan Africa? A Synthesis of the Evidence

**DOI:** 10.1080/23288604.2022.2068231

**Published:** 2022-03-01

**Authors:** Dennis Waithaka, Cheryl Cashin, Edwine Barasa

**Affiliations:** a Health Economics Research Unit, KEMRI-Wellcome Trust Research Programme, Nairobi, Kenya; b Results for Development Institute, Washington, D.C, USA

**Keywords:** Performance-based financing, results-based financing, review, strategic purchasing, Sub-Saharan Africa

## Abstract

Many countries in sub-Saharan Africa have implemented performance-based financing (PBF) to improve health system performance. Much of the debate and analysis relating to PBF has focused on whether PBF “works”—that is, whether it leads to improvements in indicators tied to incentive-based payments. Because PBF schemes embody key elements of strategic health purchasing, this study examines the question of whether and how PBF programs in sub-Saharan Africa influence strategic purchasing more broadly within country health financing arrangements. We searched PubMed, Scopus, EconLit, Cochrane Database of Systematic Reviews, Google Scholar, Google, and the World Health Organization and World Bank’s repositories for studies that focused on the implementation experience or effects of PBF in sub-Saharan African and published in English from 2000 to 2020. We identified 44 papers and used framework analysis to analyze the data and generate key findings. The evidence we reviewed shows that PBF has the potential to raise awareness about strategic purchasing, improve governance and institutional arrangements, and strengthen strategic purchasing functions. However, these effects are minimal in practice because PBF has been introduced as narrow, often pilot, projects that run parallel to and have little integration with the mainstream health financing system. We concluded that PBF has not systematically transformed health purchasing in countries in sub-Saharan Africa but that the experience with PBF can provide valuable lessons for how system-wide strategic purchasing can be implemented most effectively in that region—either in countries that currently have PBF schemes and aim to integrate them into broader purchasing systems, or in countries that are not currently implementing PBF. We also concluded that for countries to pursue more holistic approaches to strategic health purchasing and achieve better health outcomes, they need to implement health financing reforms within or aligned with existing financing systems.

## Introduction

Performance-based financing (PBF) has been one of the most studied and debated health financing approaches over the past decade. The World Health Organization (WHO) defines PBF as “a form of service provider payment where financial incentives are directed only to healthcare providers (not beneficiaries) when they achieve predetermined verified performance targets, often defined in terms of process or output indicators, adjusted by some measure of quality.”^1^ The premise is that providers exert more effort when payments are tied to specific targets or results.2 More recently, however, PBF has been put forth as a reform package that goes beyond simple provider payments to include: separation of functions between the purchaser, providers, and verifiers; increased health provider autonomy; enhanced monitoring activities; and community involvement.^2^ PBF is a subset of the umbrella term *results-based financing* (RBF), which encompasses any arrangements that link payments (monetary or in-kind) made to a government, manager, provider, payer, or beneficiary of health services when they achieve predetermined, verified results.^1,3–5^ Other labels used to refer to RBF approaches include *pay-for-performance* (P4P), *performance-based incentives* (PBI), and *performance-based payments*.^3,4^

PBF has been widely adopted in low- and middle-income countries over the past two decades, especially in sub-Saharan Africa.^3,4,6,7^ This trend has been attributed in large part to advocacy by international agencies and non-governmental organizations (NGOs).^7–10^ In sub-Saharan Africa between 2006 and 2017, the number of countries implementing PBF increased from three to 32 (out of 46), accounting for more than 2 billion USD in expenditure.^4,6,11,12^

Much of the debate and analysis relating to PBF has focused on whether PBF “works”—that is, whether it leads to improvements in indicators tied to incentive-based payments.^13–16^ Some of the literature has also focused on the effects of PBF on other indicators (aside from the indicators tied to incentives) and unintended consequences.^8,17^ Global dialogue has increasingly called for PBF to be embedded in a country’s broader health financing system, and some studies have examined the interaction of PBF with the health system more broadly.^18–20^

Because PBF schemes embody key elements of strategic health purchasing—benefits specification, contracting, provider payment, and performance monitoring^21,22^—attention has also been paid to whether PBF schemes can serve to strengthen strategic purchasing functions and systems more broadly in a country. *Strategic purchasing* refers to the allocation of resources informed, at least in part, by provider performance and population health needs.^23^ Given that PBF provides an explicit link between the delivery of prioritized services and payments based on performance data, it is increasingly seen as a tool to make purchasing more strategic.^19^ We synthesized evidence to examine whether and how PBF influences strategic purchasing within a country’s health financing arrangements. Based on the synthesized evidence, we drew lessons for countries that want to use PBF to improve strategic health purchasing more broadly.

## Methods

### Analytical Framework

**Figure 1:**
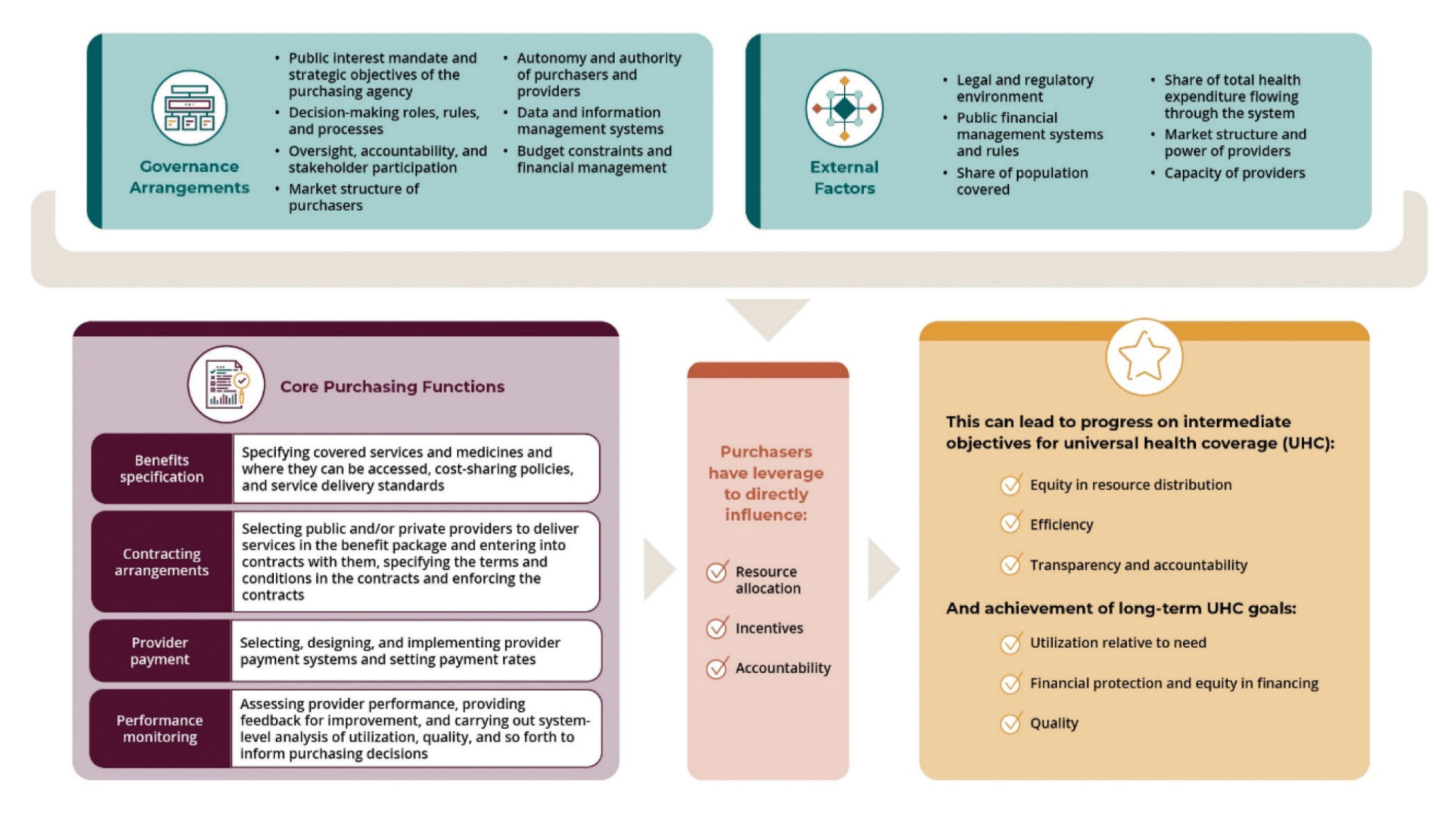
Strategic Health Purchasing Progress Tracking Framework

The analysis for this paper was guided by the Strategic Health Purchasing Progress Tracking Framework (see Figure 1). The framework focuses on the core purchasing functions of benefits specification, contracting arrangements, provider payment, and performance monitoring, as well as the governance and institutional arrangements that provide oversight and accountability for carrying out the purchasing functions effectively. The framework assumes that when purchasing functions and governance arrangements are in place, the purchaser can directly influence (positively or negatively) the allocation of resources, the incentives that influence individual provider behavior, and accountability through contract enforcement and performance monitoring. Resource allocation, incentives, and accountability can in turn affect overall progress on universal health coverage (UHC) goals. The framework also incorporates factors external to the purchasing arrangements that can either strengthen or weaken the power of purchasers to directly influence resource allocation and provider behavior, such as the share of total health funding managed by the purchaser, public financial management rules, and provider capacity.

### Literature Search

We conducted a systematic search on PubMed, Scopus, EconLit, the Cochrane Database of Systematic Reviews, Google Scholar, Google, and the WHO and World Bank repositories to identify relevant peer-reviewed and non-peer-reviewed literature. We also searched the references lists of the selected papers to identify other relevant studies. Table 1 lists the search terms we used when searching the respective databases.

### Eligibility Criteria

We included studies that met the following five criteria as suggested in Cochrane’s Effective Practice and Organization of Care (EPOC) review template.^24^ First, the study was related to the topic of interest. We included studies that synthesized evidence on at least one of the following: PBF implementation experience and intended and unintended effects of PBF. We used the term *PBF* according to WHO’s^1^ three distinguishing features of a PBF scheme: 1) incentives are directed only to providers, not beneficiaries, 2) awards are purely financial, and 3) payment depends explicitly on the degree to which providers achieve certain pre-established, verified performance targets. Second, the study was related to the study design and methods. Given the large amount of literature on PBF, we first selected systematic reviews to include in our analysis. However, we found that the majority of the reviews were either quantitative or qualitative but lacked information on the mechanisms (the how and why) through which the intended and unintended effects of PBF occurred in certain circumstances. Therefore, we also included primary studies that used qualitative study designs (such as case studies and qualitative process evaluations).^24^ We included mixed methods studies if it was possible to extract the data that were collected and analyzed using qualitative methods.^24^ We did not exclude studies based on our assessment of methodological limitations. Third, the study was published in English. English was the only language in which the review team was proficient, so this ensured that we would appropriately interpret the meaning of texts, which can be lost during translation from another language. Fourth, the study was published within the past 20 years (2000 to 2020), to focus on the most recent evidence. Fifth, were studies conducted in sub-Saharan Africa.

### Study Selection Process

**Figure 2:**
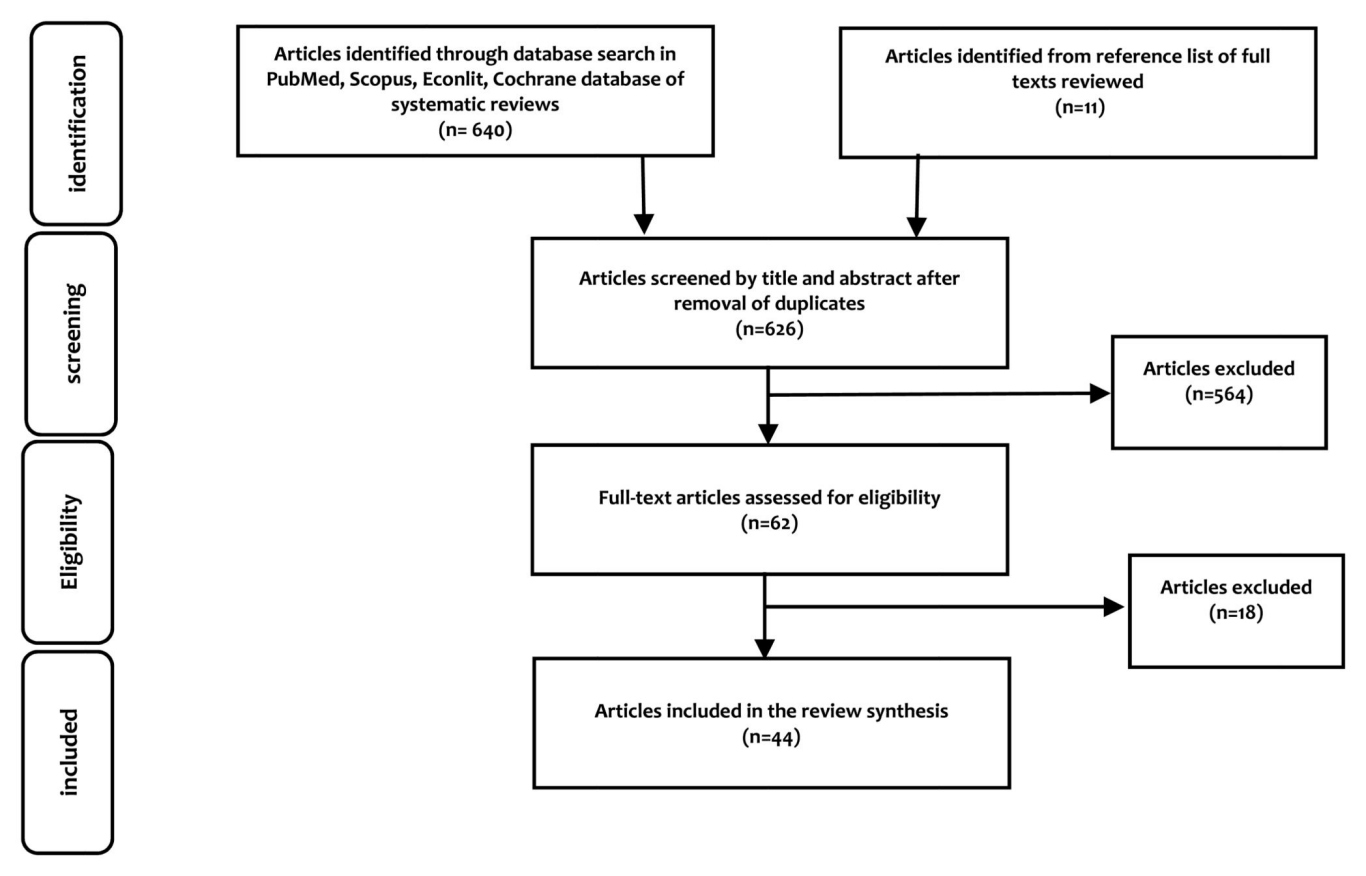
Flow chart of the study selection process (adapted from the PRISMA flow diagram)

We independently assessed the titles, abstracts, and full texts of the identified records to evaluate their eligibility. We resolved any disagreements through discussion to arrive at a consensus. We identified 651 papers, from which we removed 25 duplicates. Screening by title and abstract led to the elimination of 574 articles, and screening by reading the full papers led to the selection of 44 articles that met the eligibility criteria (see Figure 2). The key reasons for exclusion were that the study was conducted in a high-income country, it focused on demand-side incentives or nonfinancial incentives only, or it was a commentary rather than a research paper. Characteristics of the selected papers are described in Supplementary File 1.

### Data Extraction and Synthesis

We applied the framework analysis approach to generate findings and interpretations that are relevant for policy and practice.^25, 26^ We followed this analysis process: First, one author read through the selected articles to identify key themes, including those not captured in the study’s conceptual framework. Second, we generated a coding scheme informed by the conceptual framework to group results into six categories: 1) governance and institutional arrangements, 2) benefits specification, 3) contracting arrangements, 4) provider payment, 5) performance monitoring, and 6) external factors affecting the implementation and results of PBF. Third, each article was read carefully, and the relevant findings were coded. Fourth, the data were sorted and charted according to the coding scheme. Fifth, we critically examined and interpreted the charted data across articles to generate an in-depth understanding and interpretation of them.

## Results

In the following sections we synthesize the findings from the reviewed studies on whether and how PBF influences strategic purchasing within a country’s health financing arrangements in each of the domains included in the analytical framework, as well as the role of key external factors in strengthening or mitigating the role of PBF in influencing strategic purchasing more systemically.

### Strengthening Governance of Strategic Purchasing

#### Governance Structures and Institutional Arrangements

The studies included in this review suggest that in some settings, PBF can have a positive impact on governance and institutional arrangements in the health sector by defining clearer accountability frameworks, separating some key health financing functions, and clarifying roles and relationships among different actors.^27,28^ This is particularly true where health financing governance arrangements are weak, such as in post-conflict settings. One example is the Democratic Republic of the Congo (DRC), where regulatory capacity was weak at all levels (central, provincial, and zonal).^28^ PBF introduced contracts between purchasers and providers, which came with clearer rules and regulations for providers. Contracts were also signed between different levels of the Ministry of Health (MOH) hierarchy. However, these frameworks and regulations affect only PBF funding, not other funds, so it is unclear whether they can help improve governance and institutional arrangements for health purchasing more broadly.^28^

In most PBF schemes, external donors and international agencies are responsible for the purchasing of services, at least temporarily, before that function is transferred to national agencies.^29,30^ This was the case in some countries because of the assumption that the complexity of the programs exceeded the institutional capacity of national agencies.^30^ However, the evidence suggests that setting up parallel PBF agencies and operations can increase fragmentation in governance and institutional arrangements and they can be challenging to integrate into national government structures. Furthermore, even when the PBF functions are embedded in the MOH, fragmentation of institutional responsibilities can occur. In Sierra Leone, for example, the department within the Ministry of Health and Sanitation that is responsible for PBF was seen as isolated from the rest of the ministry and maintaining a bilateral relationship with donors.^31^

More recently, international partners have made explicit efforts to work with ministries of health and district health teams and leaders to avoid creating or exacerbating duplication and fragmentation. In Cameroon, for example, where PBF was launched in 2011, the purchasing of services was subcontracted to a consultancy firm and an NGO, both international, to ensure rapid implementation. But from the earliest stage, it was agreed that this role would later be transferred to a national entity.^29^ This planning from the start facilitated the transfer, as did the amendment of the legal status of the national entity so it could take on those functions.^29^

#### Health Provider Autonomy

We found some evidence in the studies we reviewed that PBF can be instrumental in introducing provider autonomy into purchasing arrangements, which can create incentives that affect provider behavior.^29, 30^ The evidence shows that for PBF incentives to be effective, providers need to have a say in management decisions, which can help them internalize and respond to the incentives. For example, when PBF was scaled up nationally in Cameroon, policies granting greater autonomy enabled facilities to creatively respond to shortages in drugs and supplies.^29^ Specifically, some facility managers took out low-interest loans from their staff to procure drugs from private retailers and prevent stockouts.^29^

In most settings in the studies reviewed, however, public financial management rules limited the autonomy and flexibility of public providers to make decisions about how to deliver services and which inputs to use. In Burundi and Malawi, for instance, PBF facilities had to make requests for drugs and staff through district health managers, using a reportedly time-consuming process that limited their autonomy and ability to respond to shortages.^32–34^ In Mozambique, public financial management laws did not allow facilities to open bank accounts, so the facilities had to rely on the district administrator to verify their expenditures and generate bank checks.^35^

### Strengthening Purchasing Functions

#### Benefits Specification

PBF specifies which service areas are tied to incentive payments, and the evidence suggests that this can help clarify which services are high priority and thereby shape the benefit package in the country’s primary coverage scheme. In Burundi, for example, PBF was used in 2006 to implement a new government policy on free health care for pregnant women and children under age five.^33^ Since PBF was implemented in Burundi, more than half of the services linked to PBF bonus payments have been included in the free services package.^33^ The PBF indicators also added additional benefits specification to the free maternal and child health—such as vaccinations for children under age one and pregnant women, four standard prenatal care visits, and institutional delivery by qualified staff.^33^

Where PBF is at least partially aligned with government-specified benefit packages, the evidence suggests that it is important to include a broad range of services to enhance buy-in. For instance, the PBF pilot in Mozambique failed to progress beyond the pilot stage partly because of its strong focus on HIV services; it thus had little buy-in as a health system reform among health workers and the MOH.^7^ Similarly, in Mali the PBF scheme focused only on three reproductive health indicators and thus was perceived as a vertical program and lacked ownership by country stakeholders.^36^ Furthermore, the evidence suggests that the benefit package should be flexible enough to address local disease outbreaks and provide locally relevant services based on local disease burdens and patterns. This was demonstrated in Zimbabwe, where the PBF scheme’s benefit package did not account for local disease patterns and outbreaks, which resulted in certain health facilities receiving relatively lower PBF bonuses when they failed to meet PBF targets because of their heavy workload related to local diseases or outbreaks.^37^

In many cases, however, the services linked to bonus payments in PBF programs are not aligned with government benefit packages in the primary coverage mechanisms, such as national health insurance systems or free care programs. For example, In the Central African Republic, DRC, and Nigeria, PBF emphasizes only some of the services in packages defined by the national government.^38^ In Sierra Leone, the PBF services have only been partially aligned with the government’s free care program.^31^ When the services included in PBF schemes do not align with the national benefit package, PBF could undermine access to services in the country’s primary coverage program. The evidence shows that health workers may focus on those targeted services over others in the package that are not tied to additional payment. This is particularly true for services that are easiest to increase in volume and therefore reap more bonus payments.^14,33,35,39–43^

#### Contracting Arrangements

In the studies reviewed, the evidence suggests that PBF can improve contracting arrangements between purchasers and health care providers when they are the first “contracts” introduced in that context. PBF contracts aim to ensure that providers are aware of the purchaser’s expectations of their performance. The contracts are typically a mutually agreed-upon document, at least between the fund holder and health care providers, which at a minimum outlines performance indicators (mostly quantity and quality of services), related payment amounts, and conditions for bonuses and sanctions.^44–48^

Contracting in PBF schemes has a greater impact on service delivery outcomes when the terms of contracts are clearly communicated and monitoring is cooperative, with the goal of supporting performance improvement.^47,49–51^ Performance feedback is important in strengthening the effects of contracting on provider performance and service delivery quality.^52^ Conversely, failure to inform providers about expectations and terms of payment affects the quality of service delivery. In Burkina Faso, providers were not well informed about how contracts, indicators, and monitoring processes were to work, leading to minimal impact on service provision.^51^

The evidence on whether PBF improves contracting as a purchasing function is varied. For instance, Witter et al. (2019)28 found that PBF improved contracting in the DRC, a country that had a weak regulatory framework for health facilities before the introduction of PBF. In countries with stronger regulatory frameworks, such as Zimbabwe and Uganda, the same study found that PBF did not have a meaningful impact on contractual arrangements in the system overall, and instead added a parallel layer of contracting.^28^ Furthermore, when PBF contracting was done outside the public sector or within the public sector but without the involvement of the national MOH, the schemes lacked national ownership and failed to progress beyond the pilot stage and therefore had minimal effect on improving contracting for health services overall.^7^

#### Provider Payment

The evidence reviewed showed that PBF can facilitate progress toward strategic purchasing by introducing payments for specific outputs in systems with traditional input-based budgets.^29–31^ This can create incentives for providers to increase productivity and improve other aspects of performance that are absent in input-based budget payment systems. Overall, however, PBF has not significantly influenced provider payment policies in most countries due to its limited scope. In Uganda, Zimbabwe, and the DRC, where PBF pilots focused on a limited set of indicators or service areas, no systemic impacts on provider payment were found.^28,53^ In all of the PBF schemes included in this review, PBF performance payments were a top-up to other mechanisms, such as facility budgets and salaries, forming a small part of overall incentives and having limited ability to encourage more systemic improvements in provider payment.

Nonetheless, evidence from PBF implementation sheds light on the many factors that affect how payment incentives affect provider behavior, which is relevant for provider payment policy more broadly. For example, the evidence from this review shows that the power of payment incentives to affect provider behavior is related to the size of payment for different services,^32,50^ the marginal cost to providers of delivering services,^32,33^ and provider workload.^49, 54^ Further, when user fees are charged, they can mute the power of payment incentives because they create a financial barrier to accessing the incentivized health care services. In Cameroon, for example, the failure of PBF incentives to improve the use of maternity services was linked to patients being unable to afford the user fees.^40^

Payment delays can also affect the power of incentives,^33,35–37,40,44,49,51,52^ with lengthy verification^33,36,44^ processes and heavy administrative burden in processing claims and making payments^35,37,44^ identified as the most common reason for payment delays in PBF schemes. In Benin’s PBF program, for example, delays of up to eight months between service provision and bonus payments were common, due to time-consuming verification processes and lengthy procedures for calculating and executing payments from the national level to facility bank accounts.^44^ In Zimbabwe, frequent delays in bonus payments were linked to “tedious” procedures for requesting and collecting bonus payments.^37^

Lack of transparency in bonus distribution has also led to perceptions of unfairness and dissatisfaction among health workers, which may affect performance and motivation.^32,51,52,55^ This was the case in Burkina Faso, where some facility managers hid the bonus amounts awarded to each health worker.^52^ Similar dissatisfaction was noted in Burundi due to lack of transparency in how much of the PBF funding was allocated to facility improvement compared to individual staff bonuses.^32^

#### Performance Monitoring

Evidence from the studies reviewed showed that PBF programs have the potential to improve the monitoring function by specifying desired service delivery targets, reporting outcomes, and verifying reported outcomes. By linking payment incentives to accurately reported information, PBF has in some cases helped to strengthen health information systems. In Benin, for example, the PBF verification process exposed data quality issues in the national health management information system (HMIS), which were addressed over a three-year period of PBF implementation.^56^

The nature of PBF supervision and verification visits can also strengthen the performance monitoring function by creating mechanisms to provide supportive performance feedback to providers. For example, in Nigeria, Cameroon, and Mozambique, even though health workers felt constantly monitored by PBF verification visits, the supportive nature of the visits and the constructive feedback motivated them to improve their performance.^47,49,50^ But verification visits can also serve a punitive function and have a negative effect on communication between purchasers and providers and reduce motivation. In Zimbabwe, Zambia, Burundi, and Burkina Faso, health workers were discouraged by the overly critical nature of the visits, which were often characterized by fault finding and lack of praise and guidance on how to improve performance.^32,33,37,51^

Overall, the evidence shows that the potential of PBF to strengthen monitoring is often limited by poor integration of PBF schemes with the rest of the health system.^28,37,44,52,53^ PBF programs often have parallel monitoring processes and sometimes parallel information systems. For example, in the DRC, the PBF scheme used a parallel information system that did not enhance the overall monitoring of service delivery in the public health system.^28^ In Zimbabwe, even though the PBF program used existing HMIS data with verification, the verified data were not fed back to the rest of the system and hence limited the contribution of PBF to strengthening the HMIS and provider monitoring systems.^28^

Furthermore, although the verification process is often carried out by actors within the public system,^57^ in many cases external agencies (such as international and local NGOs) are contracted for this function.^29–31,44,55^ The use of credible, independent agencies with highly skilled verifiers has been credited in some cases with creating the perception of greater objectivity, but it can also pose a challenge to financial sustainability, national ownership, and internal capacity building among the purchasing agencies. In Benin, for example, verification by external agencies accounted for about 50% of the cost of the PBF project, which limited the PBF program’s ability to improve governance and stewardship at the district level.^44, 55^ In Bubanza Province in Burundi, public health managers struggled to guide facilities during the nationwide scale-up of PBF because they were denied the management and leadership training needed due to the scheme’s heavy reliance on an international external agency during the pilot phase.^46^

The lack of integration of routine monitoring activities and similar activities related to PBF and other vertical programs can result in competing priorities for district managers, leading to frustrations and demotivation that affect the quantity and quality of PBF monitoring activities.^44^ The parallel verification processes in PBF schemes have been identified as a costly and burdensome approach to provider monitoring that may not bring sufficient added benefit.^44^ In Benin, for instance, district managers who were already overwhelmed and overburdened deprioritized PBF verification as too time consuming and noted that their per diem earnings were “lower and less readily paid” compared to other vertical programs.^44^ In Zimbabwe, district managers felt that their heavy responsibilities created so much pressure on their time that they could not provide quality PBF supervision.^37^

Finally, the complex and time-consuming nature of both facility and community verification processes can result in unintended consequences. For example, in Benin, it led to frustrations, poor-quality verification, and less time for other verification-related activities, such as data analysis and comprehensive feedback and coaching.^44^ In Mali, it led peer evaluators to frequently forgo verifications.^36^ In Burkina Faso, the unintended consequences of community verification included loss of patient confidentiality.^17^

### External Factors

Many factors affect the degree to which purchasing can influence resource allocation, incentives, and accountability, and in turn higher-level UHC objectives.^22^ Similarly, our study found evidence related to several key external factors that affect whether PBF programs achieve their objectives. These factors include general socioeconomic and political conditions,^33,48,58^ health system infrastructure and capacity,^32,34,37,42,45,49,52,59,60^ health provider management capacity,^29,36,37,40,52^ and cultural norms and practices.^35,37^ All of these factors are likely to be relevant for enabling system-wide strategic purchasing.

On the other hand, local adaptations during the design and implementation of PBF can facilitate creative responses to health system shocks, thereby influencing the external environment and contributing to health system resilience.^61^ For example, during the Boko Haram insurgency in Nigeria, the PBF program subcontracted with facilities to send out mobile clinics with security staff in heavily affected districts.^61^ Likewise, during the Ebola epidemic in West Africa, the PBF scheme in Guinea was adapted to include incentives for indicators related to Ebola response, such as contact tracing, notification, and confirmation of Ebola cases.^61^

#### Policy Advocacy for PBF

This review also found that the nature of policy advocacy for PBF greatly influences the enabling environment for PBF implementation as well as whether and how strategic purchasing becomes a policy priority more generally.

The enormous amount of global attention on PBF over the past decade or more, including among academic researchers, has coincided with increased awareness of strategic purchasing among policymakers in sub-Saharan African countries.^28^ This traction has been linked to strong advocacy for PBF by external actors—including international development agencies, NGOs, and international health financing experts—and has led to the introduction of PBF pilots in sub-Saharan African countries, with some subsequent scale-up to national programs and policies in some countries.^7^ National actors, interests, and advocacy have also played a role in fostering PBF introduction in some African countries.^7^ In Cameroon, for example, international donors generated interest among national health officials and influenced the degree to which PBF emerged on the national policy agenda through financial incentives, framing of PBF as a solution to poor accountability and health system inefficiency, and the creation of a cadre of local PBF experts who were able to advocate for the approach.^29^

The ability of PBF advocates to influence scaling up of PBF depended on whether PBF aligned with the political context (political interests, ideologies, and values). In Cameroon, for instance, a political focus on transparency and fighting corruption, along with existing autonomous institutions that could take on the health purchasing function, made it easier to promote the nationwide scale up of PBF.^7^ In Rwanda, PBF was more readily accepted and scaled up partly due to a pre-existing political culture that emphasized performance.^7^

However, in some cases advocacy for PBF drew the focus of policy dialogue away from broader processes of change related to strategic purchasing. This was particularly true when PBF was presented as a comprehensive approach that could address all aspects of the health system, even while being implemented at the margins.^12^ At least one study showed that the policy dialogue surrounding the introduction of PBF in sub-Saharan Africa also raised awareness about strategic purchasing more generally, although perhaps in a misleading way that equated PBF with strategic purchasing and kept the policy dialogue narrowly defined.^12,62^

## Discussion

Most of the studies reviewed shared the view that while PBF has the potential to raise awareness of strategic purchasing among country stakeholders, improve institutional and governance arrangements, and strengthen strategic purchasing functions, the effects are often limited in scope. This is because in most sub-Saharan Africa countries PBF has been introduced primarily as a separate health financing mechanism via pilot projects that run parallel to the rest of the health financing system. This limits the effects of PBF to the pilot projects rather than the broader health financing system. PBF reforms also have limited potential for uptake, integration, and impact if their introduction excludes national stakeholder involvement (and hence is viewed as an externally driven vertical program), its implementation is characterized by the setting up of parallel systems, and its design ignores the local context.^7,36,48,55^

Nonetheless, PBF programs can provide valuable lessons for how systemwide strategic purchasing can be implemented most effectively in sub-Saharan Africa. Below are lessons from PBF implementation that countries can draw on as they take steps toward implementing more holistic strategic purchasing approaches in their health systems.

- **Governance and institutional arrangements**. The evidence from PBF implementation suggests that defining clearer accountability frameworks, separating some key health financing functions, and clarifying roles and relationships among different actors can strengthen the governance arrangements for purchasing. It is also critical to embed all key purchasing functions in national institutional structures, even if some functions are outsourced in the short term. The institutional demands of the strategic purchasing functions should align with the capacity of national institutions to effectively carry them out, and the level of sophistication of purchasing arrangements can evolve as institutional capacity grows.
- **Provider autonomy**. Strategic purchasing should be accompanied by sufficient provider autonomy and leadership and management capacity to internalize and respond to the incentives created by strategic purchasing approaches and to meet the needs of the populations served.
- **Benefits specification**. Benefit packages should be aligned to avoid fragmentation and should be driven by the health priorities of the country, but flexibility should be built in to accommodate sudden changes in priorities. By adding detail within benefits specification, the purchaser can provide clearer information to providers about what is expected in terms of service delivery standards and service quality.
- **Contracting arrangements**. Contracts should be streamlined as well as clear and precise about the responsibilities of each side, the terms of payment, and the process of implementation and enforcement. Contract implementation and enforcement should facilitate providing feedback on performance rather than punitive action (unless fraud is detected).
- **Provider payment**. Payment incentives should align with service delivery objectives and send clear signals about service priorities. The context the provider is operating in should be taken into consideration when setting payment rates, including provider workload, the marginal costs to providers of delivering different services, and other policies such as cost sharing and user fees. Payment incentives should cascade from the provider institution to individual providers in a transparent way that is perceived as fair within that context. Moreover, payment processes should be administratively streamlined, to avoid payment delays and burdensome claims processes.
- **Performance monitoring**. Reporting requirements and data quality should be made explicit in contracts with providers and the monitoring systems of purchasing agencies. Monitoring information should also be shared with providers, along with supportive feedback, to enable dialogue between purchasers and providers and support performance improvement. Further, the intensity of monitoring and verification should be balanced with what it can help accomplish in terms of improved accountability and provider performance. Monitoring and verification data should be used for further system-level analysis to monitor trends, whether objectives are being met, and whether purchasing policies are leading to any unintended consequences.
- **External factors**. Aligning strategic purchasing approaches with the external environment—health system capacity, socioeconomic and geographic conditions, and cultural norms and practices—is essential. Further, strategic purchasing systems should be flexible and adaptable to changes in the context so purchasing can serve as a tool for improving health system resilience.

## Limitations

This review has two key limitations. First, restricting the eligibility criteria to studies published in English may have biased our findings. However, this was partly mitigated by the inclusion of reviews whose own eligibility criteria were not necessarily limited to studies published in English. Second, most of the included studies did not focus on strategic purchasing, which means that several aspects of strategic purchasing were not examined or discussed in detail. This affected the depth of discussion in our review findings as well.

## Conclusions

Much of the debate and analysis relating to PBF has focused on whether and how PBF “works” and has thus been of limited value in informing policy dialogue at the country level. These debates and analyses have provided little insight into how PBF can make health financing systems in general, and strategic purchasing in particular, more effective. Many countries in sub-Saharan African countries have implemented PBF, but it has largely been implemented at the margins of the health financing system and has not been transformative.

Nonetheless, PBF programs have served as an entry point to strategic purchasing approaches, in some countries introducing the tools of contracting and output-based provider payment for the first time. The experience with PBF can provide valuable lessons for how system-wide strategic purchasing can be implemented most effectively in sub-Saharan Africa— either in countries that currently have PBF schemes and aim to integrate them into broader purchasing systems, or in countries that are not currently implementing PBF. The experience with PBF also makes clear, however, that in order to pursue more holistic approaches to strategic health purchasing and achieve better health outcomes, health financing reforms need to be embedded in and aligned with existing financing systems and the broader context of the country.

## Supplementary Material

Supplemental material for this article can be accessed online at https://doi.org/10.1080/23288604.2022.2068231

Supplemental material

## Figures and Tables

**Table 1:** Literature search: databases, dates of final search, and search terms.

Database	Date of Final Search	Search Terms
PubMed	22/02/2020	(systematic review OR review OR meta-analysis) AND (“performance based financing” OR pbf OR “pay for performance” OR p4p OR “performance based payments” OR “performance based incentives” OR “results based financing” Or RBF OR “paying for results” OR “paying for performance” OR “value based purchasing”) AND (“developing countries” OR “low- and middle-income countries” OR “low income countries” OR africa)
Scopus	22/02/2020	(TITLE-ABS-KEY (“systematic review”) OR TITLE-ABS-KEY (review) OR TITLE-ABS-KEY (meta-analysis) AND TITLE-ABS-KEY (“performance-based financing”) OR TITLE-ABS-KEY (“pay for performance”) OR TITLE-ABS-KEY (“performance based payments”) OR TITLE-ABS-KEY (“performance based incentives”) OR TITLE-ABS-KEY (“results based financing”) OR TITLE-ABS-KEY (“paying for results”) OR TITLE-ABS-KEY (“paying for performance”) OR TITLE-ABS-KEY (value AND based AND purchasing) AND TITLE-ABS-KEY (“developing countries”) OR TITLE-ABS-KEY (“low- and middle-income countries”) OR TITLE-ABS-KEY (“low income countries”) OR TITLE-ABS-KEY (africa) AND (LIMIT-TO (DOCTYPE, “re”)) AND (LIMIT-TO (LANGUAGE “English”))
EconLit	22/02/2020	(systematic review OR review OR meta-analysis) AND (“performance based financing” OR pbf OR “pay for performance” OR p4p OR “performance based payments” OR “performance based incentives” OR “results based financing” OR RBF OR “paying for results” OR “paying for performance” OR “value based purchasing”) AND (“developing countries” OR “low- and middle-income countries” OR “low income countries” OR africa)
Cochrane Database of Systematic Reviews	22/02/2020	(“performance-based financing” OR “pay-for-performance” OR “performance-based payments” OR “performance-based incentives” OR “paying-for-results” OR “results-based financing”)
