# Supplementary material for: Is Performance-Based Financing A Pathway to Strategic Purchasing in Sub-Saharan Africa? A Synthesis of the Evidence
Source: Health Syst Reform. Author manuscript; Available in PMC 2022 Sep 6. (PMC7613548; doi:10.1080/23288604.2022.2068231)
Supplement: Supplemental material [file EMS153580-supplement-Supplemental_material.docx]

**Supplementary File 1:**

**Characteristics of the Selected Papers**

***Systematic Reviews***

The following table presents the characteristics of the selected reviews. We included only reviews that contained studies done in sub-Saharan Africa. The majority of the papers were quantitative (eight out of 15), but seven out of the eight quantitative papers did not conduct statistical pooling (meta-analysis) because of the heterogeneity in PBF design, implementation and context, and heterogeneity of individual study designs and analysis. For similar reasons, this review also did not conduct a meta-analysis. Most of the reviews analyzed the findings using narrative synthesis (12 out of 15), with a few using summary measures. Most of the reviews focused mainly on the effectiveness of PBF (10 out of 15), with the rest focusing on the cost-effectiveness of PBF, how PBF works, and the factors influencing the PBF policy process, including the role of context. The median number of individual studies and documents included in each review was nine, with a range of four to 140 documents. Most of the individual study designs were observational and were conducted in Rwanda. Most of the individual studies were published within the past 15 to 20 years, which shows that this a growing area of interest. Where indicated in the review, the individual studies that examined the effects, effectiveness, or cost-effectiveness of PBF lasted between 1.6 and 3.2 years (short-term). The study participants in most of the individual studies were health facilities, and the PBF schemes mainly targeted maternal and child health services.

**Table 1. Characteristics of the Selected Reviews**

| **Review Citation** | **Objective of Review** | **Type of Review** | **Quality of the Review** | **Method of Synthesis** | **Main Focus** | **Number of Studies** | **Type of Studies*** | **Country Focus** | **Publication Date Range of Studies** | **Average Duration of Studies (Years)** | **Study Participants** | **PBF-Targeted Services** |
| --- | --- | --- | --- | --- | --- | --- | --- | --- | --- | --- | --- | --- |
| Witter et al.^[14]^ | To assess current evidence on the effects of PBF on the provision of health care and health outcomes in low- and middle-income countries (LMICs) | Quantitative | High | Narrative synthesis plus summary measures, such as risk ratios and regression coefficient (no meta-analysis done) | Effectiveness of PBF | 9 | Randomized controlled trial (RCT) (1), controlled before-after (CBA) (6), interrupted time series (ITS) (2) | Rwanda (2), Burundi (1), Democratic Republic of the Congo (DRC) (1), Tanzania (1), Zambia (1), Philippines (1), Vietnam (1), China (1) | 2003–2010 | 1.8 | Health facilities | Wide range of services, with a strong focus on maternal and child health (MCH) services |
| Turcotte-Tremblay et al.^[64]^ | To identify and synthesize existing literature that examines whether PBF represents an efficient manner of investing resources in LMICs (cost-effectiveness) | Quantitative | High | Summary measures such as technical efficiency scores, Malmquist productivity index, difference in costs (no meta-analysis done) | Cost-effectiveness of PBF | 7 | RCT (1), observational (6); no specific design named | Rwanda (3), Afghanistan (1), Belize (1), Haiti (1), Turkey (1) | 2006–2015 | 2.7 | Health facilities | Not specified |
| Blacklock et al.^[65]^ | To review evidence on the effectiveness of PBF in increasing contraception uptake and continuation in LMICs | Quantitative | High | Narrative synthesis (no meta-analysis done) | Effectiveness of PBF in improving contraceptive services | 13 | RCT (3), CBA (9), ITS (1) | Rwanda (6), Burundi (2), DRC (2), Tanzania (1), Nicaragua (1), Afghanistan (1) | 2005–2016 | 2.5 | Health facilities | MCH services |
| Yuan, et al. ^[66]^ | To assess the impact of different payment methods on the performance of outpatient care facilities and to analyze differences in the impact of payment methods in different settings | Quantitative | High | Narrative synthesis, summary measures, and meta-analysis; 5 of the 8 LMICs excluded from the analysis | Effectiveness of PBF | 7 | RCT (2), CBA (5) | Rwanda (1), Burundi (3), Tanzania (1), DRC (1), Afghanistan (1), China (1) | 2008–2016 | Not indicated | Health facilities | Majority focused on MCH services, with one each targeting chronic services and antibiotic prescription |
| Suthar et al. ^[16]^ | To review evidence on the effects of PBF on HIV/AIDS service delivery | Quantitative | Low | Summary measures, specifically relative risks (no meta-analysis done) | Effectiveness of PBF in improving HIV/AIDS services | 4 | RCT (1), observational (3); no specific design named | Rwanda (1), Cote d’Ivoire (2), Kenya (1) | 2009–2013 | 1.6 | Health facilities | HIV/AIDS services |
| Renmans et al.^[54]^ | To summarize current knowledge on how PBF works | Qualitative | Low | Narrative synthesis | How PBF works | 35 | Not indicated (mostly qualitative studies) | Rwanda (14), Burundi (7), Tanzania (6), Cambodia (3), DRC (2), Benin (1), Uganda (1), Cameroon (1) | 2006–2015 | Not indicated | Not specified | Not specified |
| Das et al.^[15]^ | To review existing evidence on the effects of PBF programs on quality of MCH care in LMICs | Quantitative | Critically low | Summary measures such as odds ratio and regression coefficient (no meta-analysis done) | Effectiveness of PBF, specifically in improving the quality of MCH services | 8 | RCT (3), CBA (4), case control (1) | Philippines (2), DRC (2), Burundi (2), Rwanda (1), Egypt (1) | 2010–2014 | Not indicated | Health facilities | MCH services |
| Eichler et al.^[39]^ | To review the evidence on the impact of PBF on the quantity and quality of health services for mothers and newborns | Quantitative | Critically low | Narrative synthesis (no meta-analysis done) | Effectiveness of PBF in improving MCH services | 9 | RCT (1), CBA (4), before-after with no controls (3), time series (1) | Rwanda (1), DRC (1), Egypt (1), Afghanistan (1), Cambodia (1), Haiti (1) Bangladesh, Nepal (1), Philippines (1) | 2009–2011 | 3.2 | Health facilities | MCH services |
| Bellows et al.^[67]^ | To identify and summarize the existing evidence on the effectiveness of performance-based incentives in community-based family planning programs | Quantitative | Critically low | Narrative synthesis | Effectiveness of PBF in improving community-based family planning programs | 6 | Cross-sectional comparison (1), before-after with no controls (1), qualitative studies (2), design not indicated (2) | Taiwan (1), India (1), Philippines (1), Columbia (1), Thailand (1) Bangladesh (1) | 1972–2003 | Not indicated | Community-based health workers | Contraceptive use in communities |
| Gorter and Meessen^[68]^ | To review how PBF applies to maternal and neonatal health care and the effects of the reviewed schemes on the performance of health care providers | Qualitative | Critically low | Narrative synthesis | Effectiveness of PBF on MCH services | Number of PBF-only studies not clearly indicated | Not specified for most studies | Not specified for PBF studies, only that most were from Africa | Not indicated | Not indicated | Health facilities | Essential services, with a focus on MCH |
| Eldridge and Palmer^[13]^ | To systematically review of the literature on PBF payments | Qualitative | Critically low | Narrative synthesis | Effectiveness of PBF | 27 | Not indicated | Not indicated | Not indicated | Not indicated | Not specified | Not specified |
| Shroff et al.^[7]^ | To identify the enablers of and barriers to the scale-up of PBF programs | Qualitative | Critically low | Narrative synthesis | Factors influencing the PBF policy process | 10 | Not indicated | Rwanda (1), Burundi (1), Cameroon (1), Chad (1), Mozambique (1), Kenya (1), Uganda (1), Tanzania (1), Armenia (1), Cambodia (1) | 2015 | Not indicated | Not specified | Health indicators that mostly focus on MCH services |
| Bertone, et al.^[62]^ | To review existing grey and published literature on how the context of fragile and conflict-affected (FCAS) states affects the adoption, adaptation, implementation, and health system effects of PBF, in order to support or refute a set of hypotheses about their interaction | Qualitative | Critically low | Hypothesis-led narrative synthesis | How FCAS context influences the PBF policy process | 140 documents | Academic (published and unpublished), operational manuals and research reports | DRC, Burundi; Rwanda, Central African Republic, Mali, Cambodia, Cameroon, Lao PDR, Afghanistan, Comoros, Congo, Sierra Leone, Zimbabwe, Nigeria, Liberia, The Gambia, Cote d’Ivoire, Chad, Nigeria, Haiti, Tajikistan, Djibouti, Guinea Bissau | Not indicated | Not indicated | Not specified | Not specified |
| Toonen, et al.^[48]^ | To identify lessons on the contributions to health service improvements, including the positive and negative effects of PBF | Qualitative | Critically low | Narrative synthesis | Effectiveness of PBF | Not clear | Not indicated | Rwanda, Burundi, Tanzania, Zambia | Not indicated | Not indicated | Not specified | Majority focused on MCH services; a few included HIV/AIDS services |
| Miller and Babiarz^[43]^ | To examine experiences with PBF in developing country health programs | Qualitative | Critically low | Narrative synthesis | Not clear | Not clear | Not indicated | Not indicated | Not indicated | Not indicated | Not specified | Not specified |

***Qualitative Papers***

Table 2 presents the characteristics of the 29 empirical papers included in the review. Most of the studies were qualitative (n=23), with six adopting mixed-methods study design. Most of the studies focused on either how PBF affected health worker motivation (n=8) or various aspects of the PBF implementation process (n=8). Most of the individual country studies were from Burkina Faso (n=4). Most of the study participants were health care providers (health workers and health managers), and the most commonly PBF targeted health care services were maternal and child health services.

**Table 2. Characteristics of the Selected Empirical Papers**

| **Study Citation** | **Objective** | **Area of Focus** | **Study Design** | **Country/Region** | **Study Participants** | **PBF-Targeted Services** |
| --- | --- | --- | --- | --- | --- | --- |
| Lohmann et al.^[60]^ | To investigate whether and how PBF affected intrinsic motivation | How PBF affected health worker motivation | Mixed methods | Malawi | Health care providers | Maternal and newborn health services |
| Lohmann et al.^[34]^ | To explore how PBF affected health worker motivation | How PBF affected health worker motivation | Qualitative | Malawi | Health care providers | Maternal and newborn health services |
| Kambala et al.^[59]^ | To assess the effects of PBF on quality of care from women’s perspectives | Perspectives on PBF’s effect on quality | Mixed methods | Malawi | Health care providers and patients | Maternal and newborn health services |
| Turcotte-Tremblay et al.^[17]^ | To document the unintended consequences of community verification | Unintended effects of community verification | Qualitative | Burkina Faso | Health care providers, community verifiers, and patients | Maternal and child health (MCH) services |
| Ye et al.^[41]^ | To explore health care provider preferences for an incentive scheme based on local resources | Health providers’ incentive preference | Qualitative | Burkina Faso | Health care providers | MCH services |
| Ridde et al.^[51]^ | To analyze PBF implementation in Burkina Faso 12 months post‐launch in late 2014 | PBF implementation | Qualitative | Burkina Faso | Health care providers, patients, and community | MCH services |
| Fillol et al.^[52]^ | To investigate how health workers’ perceptions of preexisting organizational factors and PBF shape their motivations | How context and PBF influence health worker motivation | Qualitative | Burkina Faso | Health care providers | MCH services |
| Antony et al.^[44]^ | To explore the implementation of a PBF pilot by focusing on the verification of results | PBF implementation, with a focus on verification | Mixed methods | Benin | Health care providers, PBF technical assistants, and community verifiers | Essential services, with a strong focus on MCH services |
| Paul et al.^[55]^ | To analyze how two PBF approaches were piloted in Benin and what effects they produced | PBF implementation | Qualitative | Benin | Health care providers | MCH services |
| Paul et al.^[56]^ | To analyze and draw lessons from the experience of the Belgian Development Agency–supported PBF alternative approach developed in Benin, taking into account the context and exploring the mechanisms used to better understand how the “PBF package” functions and produces its effects | PBF implementation and effects | Mixed methods | Benin | Donors, health care providers, and technical assistants | MCH services |
| Shen et al.^[45]^ | To examine the effects of PBF on health worker job satisfaction, motivation, and attrition | How PBF affected job satisfaction, motivation, and attrition | Mixed methods | Zambia | Health care providers | MCH services |
| Gergen et al.^[50]^ | To explore perceived changes to internal and external drivers of health worker motivation associated with PBF | How PBF influences health worker motivation | Qualitative | Mozambique | Health care providers | MCH and HIV/AIDs services |
| Schuster et al.^[35]^ | To describe PBF implementation and assess how PBF affects the workplace environment and health worker motivation, satisfaction, and thoughts of leaving their position | PBF implementation and how it affects health worker motivation | Mixed methods | Mozambique | Health care providers | MCH and HIV/AIDs services |
| Chimhutu et al.^[42]^ | To explore experiences of care from the perspectives of health workers, service users, and community health governing committee members | Perspectives on the effects of PBF | Qualitative | Tanzania | Health care providers, patients, and community | MCH services |
| De Allegri, et al. ^[40]^ | To investigate factors that may explain variation in performance across PBF indicators and facilities | How and why PBF had mixed results | Qualitative | Cameroon | Health care providers and community | MCH services |
| Sieleunou et al.^[47]^ | To explore how PBF in Cameroon affected the availability of essential medicines and to understand the mechanisms involved | How PBF influenced drug availability | Qualitative | Cameroon | Health care providers and community | MCH services |
| Bertone and Meessen^[46]^ | To propose an analytical framework for looking at institutional arrangements and to confirm its usefulness by showing how it can provide insights into the mechanisms of PBF in Burundi | PBF implementation | Qualitative | Burundi | Health care providers | Essential services, with a strong focus on MCH services |
| Rudasingwa and Uwizeye^[32]^ | To elicit physician and nurse experiences and views on how PBF influenced and helped them in health care delivery | Perspectives on the effects of PBF | Qualitative | Burundi | Health care providers | Essential services, with a strong focus on MCH |
| Nimpagaritse et al.^[33]^ | To understand change mechanisms triggered by PBF, focusing on the introduction of malnutrition prevention and care “indicators” within the preexisting PBF-free health care program in Burundi | How context and implementation influenced PBF programs | Qualitative | Burundi | Health care providers | Nutritional services |
| Bhatnagar and George^[49]^ | To understand changes in perceived motivation among health workers with the introduction of PBF in the Wamba District of Nigeria | How PBF influenced health worker motivation | Qualitative | Nigeria | Health care providers | MCH services |
| Kane et al.^[37]^ | To understand why health workers working under the PBF arrangements in Zimbabwe reported being satisfied with the improvements in working conditions and compensation but paradoxically reported lower motivation levels compared to those not working under PBF arrangements | How and why PBF influenced health worker job satisfaction and motivation | Qualitative | Zimbabwe | Health care providers | MCH services |
| Feldacker et al.^[61]^ | To explore the effects of PBF incentives on Zimbabwe’s voluntary male medical circumcision program | Perspectives on the effects of PBF | Qualitative | Zimbabwe | Health care providers | Male medical circumcision |
| Witter et al.^[53]^ | To examine how PBF affects health care purchasing | Effects of PBF on health care purchasing | Qualitative | Zimbabwe | Health care providers | MCH services |
| Witter et al.^[28]^ | To examine the effects of several PBF programs on health care purchasing functions in three fragile, post-conflict settings—Uganda, Zimbabwe, and the DRC—over the previous decade | Effects of PBF on health care purchasing | Qualitative | Zimbabwe, Uganda, Democratic Republic of Congo | Health care providers | MCH services |
| Zitti et al.^[36]^ | To analyze the process of implementing PBF at the district hospital level | PBF implementation at hospital level | Qualitative | Mali | Health care providers and community | MCH services |
| Bertone et al.^[30]^ | To examine why and how PBF has emerged and been adapted to unsettled and dynamic contexts, what the opportunities and challenges have been, and what lessons can be drawn | Interaction of PBF with contexts | Qualitative | Northern Nigeria, Central African Republic, and South Kivu in the DRC | Health providers, implementation agencies, and PBF consultants | Varied, depending on the three countries national basic packages, including MCH services |
| Bertone et al.^[31]^ | To explore decision-making processes for PBF in Sierra Leone during the 2010–2017 period | PBF policy introduction | Qualitative | Sierra Leone | Health providers, donors, local partners, and technical advisers | MCH services |
| Jacobs et al.^[38]^ | To examine whether and how PBF is aligned and integrated with national health financing strategies, particularly in fragile and conflict-affected settings | Interaction of PBF with the context, specifically national health financing strategies | Qualitative | Central African Republic, DRC, and Nigeria | Health providers, consultants, implementing and purchasing agencies | National basic package of health services |
| Gautier et al.^[12]^ | To analyze the construction of the global discourse on PBF | PBF policy discourse | Qualitative | Sub-Saharan Africa | Academics, policy makers, PBF consultants, implementing organizations | Not specified |
